# Eco-Evolutionary Feedback and the Invasion of Cooperation in Prisoner's Dilemma Games

**DOI:** 10.1371/journal.pone.0027523

**Published:** 2011-11-18

**Authors:** Feng Zhang, Cang Hui

**Affiliations:** Centre for Invasion Biology, Department of Botany and Zoology, Stellenbosch University, Matieland, South Africa; University of Zürich, Switzerland

## Abstract

Unveiling the origin and forms of cooperation in nature poses profound challenges in evolutionary ecology. The prisoner's dilemma game is an important metaphor for studying the evolution of cooperation. We here classified potential mechanisms for cooperation evolution into schemes of frequency- and density-dependent selection, and focused on the density-dependent selection in the ecological prisoner's dilemma games. We found that, although assortative encounter is still the necessary condition in ecological games for cooperation evolution, a harsh environment, indicated by a high mortality, can foster the invasion of cooperation. The Hamilton rule provides a fundamental condition for the evolution of cooperation by ensuring an enhanced relatedness between players in low-density populations. Incorporating ecological dynamics into evolutionary games opens up a much wider window for the evolution of cooperation, and exhibits a variety of complex behaviors of dynamics, such as limit and heteroclinic cycles. An alternative evolutionary, or rather succession, sequence was proposed that cooperation first appears in harsh environments, followed by the invasion of defection, which leads to a common catastrophe. The rise of cooperation (and altruism), thus, could be much easier in the density-dependent ecological games than in the classic frequency-dependent evolutionary games.

## Introduction

Cooperation within and between species abounds in nature ranging from microbial interactions to the mutualistic behavior of animals and humans [1], [2], [3], [4], [5], [6], [7], [8], [9]. Cooperative individuals can benefit others at cost to themselves, and can be easily exploited by selfish individuals that only receive benefit without cost (or contribution). As such, cooperation seems incompatible with Darwinian natural selection. However, mutual cooperation can often produce higher benefit than costs for both actors. It is, thus, necessary to seek mechanisms underpinning such a social dilemma [10] and the cooperation evolution.

Multiple hypotheses have been proposed for cooperation to initialize and sustain in selfish populations, including kin selection [11], [12], [13], group selection [14] and reciprocal altruism [15]. The most fundamental requirement for the evolution of cooperation is to break the random interaction among individuals and to construct assortative encounters between cooperative individuals [16], [17]. Assortative interactions can guarantee a close relatedness between the actor and recipient, and thus ensure the satisfaction of the Hamilton rule [12], stating that cooperation can be favored by natural selection if the benefit to cooperate, after discounted by the relatedness between players, is still larger than the cost [12], [18]. The Hamilton rule portrays the general condition for the evolution of cooperation and has been confirmed under different altruistic mechanisms [17], [19], [20].

Cooperative behaviors in evolutionary games can be categorized into two groups: (i) those that benefit both the recipient and the actor and (ii) those that benefit only the recipient [21], [22]. The former is often formulated by the snowdrift game for pairwise interactions [23] and by the public good game for group interactions [24], [25], [26], whilst the latter by the prisoner's dilemma game (PDG) [27]. The analyses of these classic evolutionary games, for instance using replicator equations, often assume infinite or constant population size for simplicity, and reflect the frequency-dependent selection [28], [29], [30]. This assumption inevitably ignores the population dynamics.

However, mounting evidence indicates that ecological and evolutionary dynamics could be commensurate in time and interact in a feedback loop [31]. Specifically, the evolution of cooperation can be facilitated by ecological factors such as the demographic stochasticity [32], [33], [34], empty sites [35], greater frequency of catastrophes [36], moderate habitat destruction and fragmentation [37], [38], [39] and intermediate disturbance [40]. In return, this behavioral evolution can also affect the dynamics and persistence of populations [37], [38], [39]. These results suggest that the density-dependent selection, in contrast to the frequency-dependent selection, could promote the evolution of cooperation [41].

The exclusion of cooperation in the social dilemma can be ascribed to the exploitation of cooperators by defectors without compensation. By reducing the population density and, thus, the encounter probability, cooperators are able to mitigate the exploitation by defectors. Consequently, the population density will climb due to accumulated benefits from mutual cooperation, which in turn begets the revival of defection and, consequently, the decline of population density, forming an eco-evolutionary feedback cycle. Such interplay between ecological forces and evolutionary games can foster the coexistence of cooperators and defectors in a public goods game, in which cooperation benefits both the recipient and the actor [42], [43], [44]. Here, we focus on the prisoner's dilemma games, in which cooperation only benefits the recipient, not the actor itself (i.e. an altruistic behavior).

To reveal how and to what extent the population dynamics and the eco-evolutionary feedback loop can affect the evolution of cooperation, we here examine the dynamics of an ecological PDG using an extension of replicator equations. Our model distinguishes two life-history stages in the population (i.e. interaction and dispersion) and assumes that the interactions (or games) between individuals happen locally and natal dispersal globally [45]. An enhanced relatedness between locally interacting individuals could rise from delayed natal dispersal (e.g. cooperative breeding) [46], [47], [48] or sibling-coalition dispersal [49], [50]. Our model differs from those for viscous populations where distances of interaction and offspring movement are considered equal [51], [52], [53]. In this study, we focus on the invasion dynamics of cooperation and the alteration of invasion condition in ecological games, and emphasize that cooperation can be promoted by the enhanced relatedness between players rising from the ecological dynamics. Using the stability analysis and bifurcation on phase planes, we portray the entire landscape of the evolutionary dynamics of cooperation in ecological PDGs.

## Analysis

### Ecological Prisoner's Dilemma Games

A cooperator (C) can produce a benefit of *b* to its recipients at a personal cost of *c* (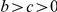), whereas a defector (D) produces no benefit and bears no cost. This derives the following payoff matrix for the Prisoner's Dilemma game (PDG) [23]:
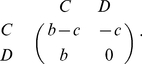
(1)Following Eshel and Cavalli-Sforza [16], we define the assortative encounter as: (i) an individual interacts with individuals of the same-strategy at a probability of *m*, and (ii) randomly plays game with other individuals, including those from the same strategy, at a probability of 1−*m*. Thus, in a population with a proportion of *x* cooperators and *y* defectors, the mean payoff for a cooperator is 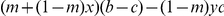 and for a defector 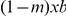. Specifically, assortment is a necessary condition for the evolution of cooperation in the ecological context, which could be imposed by (i) delayed natal dispersal [46], [47], [48], (ii) sibling-coalition dispersal [49], [50], or (iii) kin recognition [54]. In the frequency-dependent selection [28], [29], population size is constant, 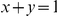; in the density-dependent selection here, treating *x* and *y* as the proportions of habitat occupied respectively by cooperators and defectors, population size is variable according to ecological dynamics, 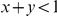.

To incorporate population dynamics into an evolutionary PDG, we consider a habitat consisting of suitable sites; each site can be occupied by either a cooperator or a defector, or remain empty. An empty site can become occupied by a new individual, whereas the death of an individual leaves its dwelling site empty. An individual is assumed to only produce offspring with the same strategy, which go on to randomly seek empty sites via global natal dispersal for colonization. The population dynamics can be described by the following differential equations [42],
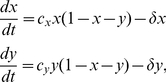
(2)where 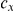 and 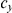 are the birth rate of cooperators and defectors, respectively; 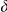 denotes the death rate (i.e. mortality). The density-dependent selection is incorporated in this ecological PDG because each individual has a probability 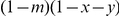 of failing to encounter other players. Following van Baalen and Rand [51], we let the birth rate be density dependent and equal a baseline reproduction rate (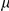) plus the payoff from playing games with others:
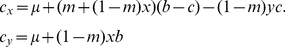
(3)It is also worth noting that, by keeping the birth rate 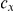 and 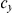 constant, this model becomes Levins' [55] metapopulation model [56]. By setting a frequency-dependent death rate 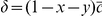 (where 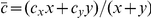 indicates the mean fitness of the population), this model turns into the evolutionary replicator equations [28], [42]. Clearly, our model of ecological PDG not only represents the ecological dynamics of birth and death (i.e. the colonization and extinction in metapopulations), but also the dynamics of evolutionary games in animals. We, thus, explored the invasion condition of cooperation, as well as the complicated dynamics of the eco-evolutionary feedback, in this ecological PDG model.

### Invasion Condition

When there is no assortment among individuals (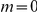), the cooperative strategy cannot invade a defective population, whereas the defective strategy can invade a cooperative population due to the initial condition 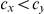. This defines the social dilemma, and suggests that assortment is a necessary condition for the cooperation evolution in the ecological PDG. When there is assortment among individuals (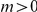), the condition for cooperation to increase in the population can be obtained by the inequality 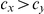, which gives:
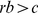
(4)where *r* represents the probability for a cooperator to have a game with another cooperator (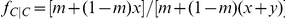) minus the probability for a defector to have a game with a cooperator (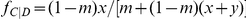); that is, 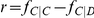. Let *X* and *Y* be the random variables of the states of the actor and recipient ( = 1 for cooperator and  = 0 for defector), then *r* is equal to the covariance between *X* and *Y*, *Cov*(*X*,*Y*), divided by the variance of *X*, *Var*(*X*) (see the detail deduction in Appendix S1):
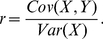
(5)This is a widely-used measure of the relatedness [18], [53], [57], and therefore, the inequality (4) of our system represents the Hamilton rule (*rB*>*C*) [12], [18], where *r*, *B* ( = *b*) and *C* ( = *c*) represent the relatedness, fitness benefit and fitness cost, respectively. Under the frequency-dependent selection (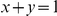), we derive that the relatedness is equal to the assortment (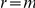). However, under the density-dependent selection (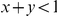), we have 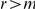. Moreover, the relatedness (*r*) increased with the decrease of population density (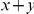). As according to the Hamilton rule (eqn. 4), cooperation evolution becomes easier (i) under density-dependent selection and (ii) especially when the population density is low.

When the proportion of cooperators in the population is initially trivial (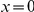), the inequality (4) can be rewritten as:
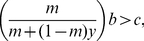
(6)Clearly, the cooperation strategy can easily invade a defective population if the density of the defective population (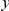) is low. Even if a pure-defector population cannot persist (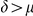), cooperators can still colonize the empty habitat if the death rate was relatively low 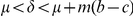. When the death rate was moderate 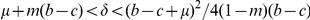, the colonization by cooperators can be successful only if the proportion of initial cooperators reaches a threshold (i.e. an Allee effect [58]),
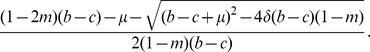
When the death rate was too high (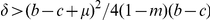) even cooperators cannot be sustained.

Once the cooperators have established themselves in the empty habitat, the invasion of defectors, in turn, can become possible if
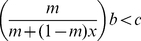
(7)This inequality implies that a cooperative population with low density can prevent the invasion of defectors. When both inequality (6) and (7) were satisfied, the mutual invasion of cooperators and defectors can lead to the coexistence of these two strategies, and potentially incur complicated population dynamics (see below). This interesting parameter range of complicated dynamic behaviors does not exist in the frequency-dependent selection (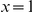 or 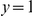), leading to the Hamilton rule of 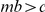 for the invasion of cooperation and 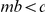 for the invasion of defection.

### Evolutionary Dynamics

There were at most two boundary equilibriums (i.e. pure strategy) in the system (eqn 2): (i) two boundary equilibriums if 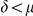; one for pure defection and the other for pure cooperation; (ii) no boundary equilibrium for defectors and only one boundary equilibrium for cooperators if 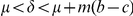; (iii) two boundary equilibrium for cooperators if 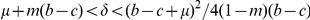. The system has at most one interior equilibrium; it depicts the coexistence of cooperators and defectors and appears when the following condition is met:
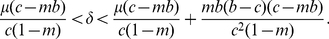
(8)No interior equilibrium exists if 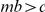. The interior equilibrium changed from a stable node to a focus when the eigenvalue of the Jacobian matrix changed from a negative real number to an imaginary number. Furthermore, the interior equilibrium can become unstable and lead to a limit cycle, determined by the Hopf bifurcation (Appendix S2). When there were two boundary equilibriums for cooperators, a heteroclinic bifurcation occurred once the limit circle had touched the unstable boundary equilibrium (Appendix S2), breaking the local stable structure on the phase plane and causing population extinction (Animation S1).

**Figure 1:**
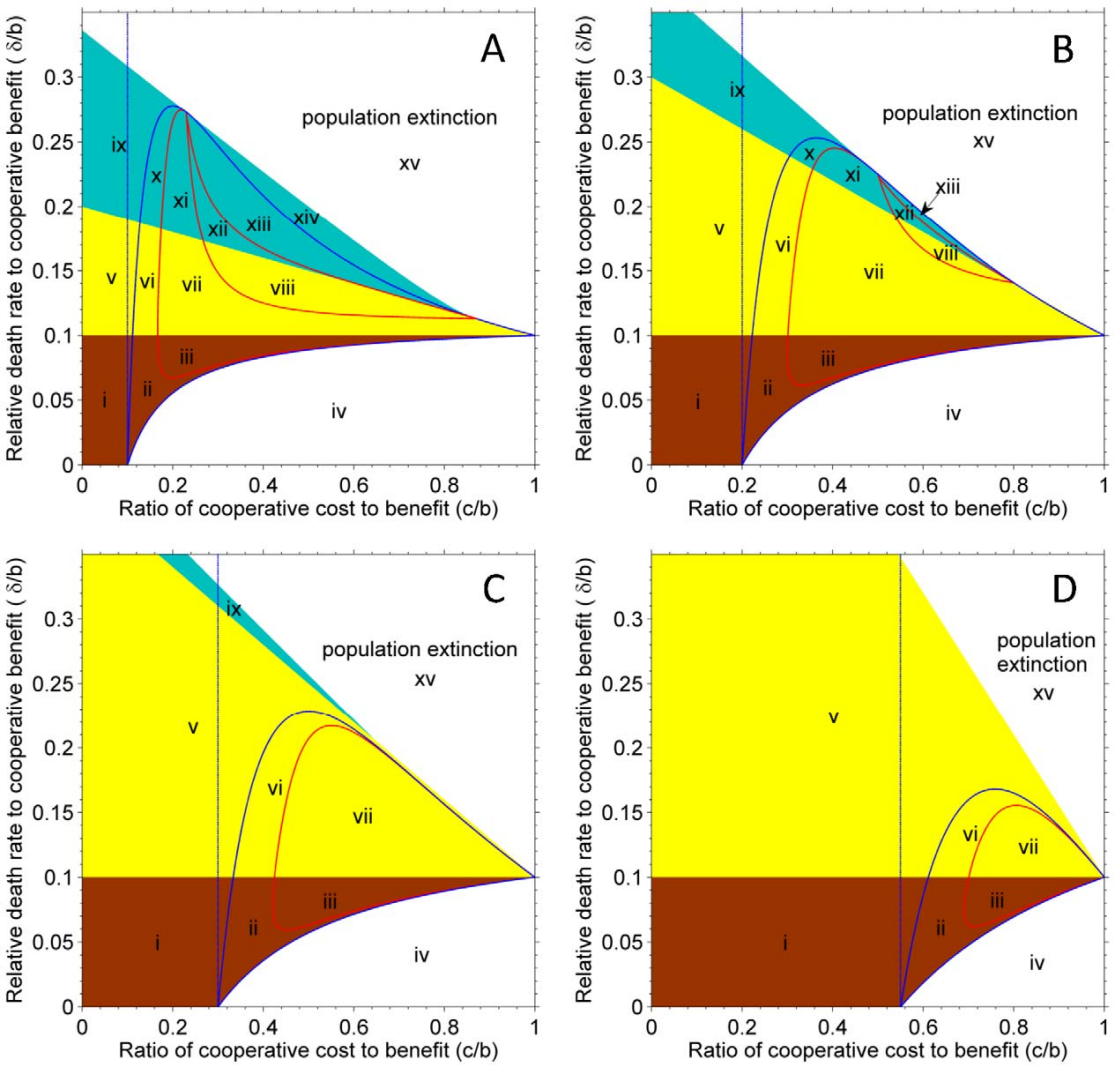
The dependence of dynamical behaviors on model parameters. Brown part and part iv, 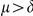, have two boundary equilibriums: one for cooperators; the other for defectors. Yellow part, 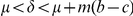, has only one boundary equilibrium for cooperators. Cyan part, 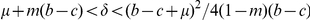, has two boundary equilibriums both for cooperators. The area encircled by blue curves indicates the existence of interior equilibrium of cooperation-defect coexistence. The three red lines on (A) and (B), from bottom to top, indicate the node-focus bifurcation, Hopf bifurcation and the heteroclinic bifurcation, respectively, and the red lines on (C) and (D) indicate the node-focus bifurcation. Area on the left side of the vertical dotted line indicates the invasion condition for cooperation in the frequency-dependent selection (i.e. 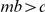). Parameters are 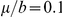 and *m* = 0.1, 0.2, 0.3 and 0.55, respectively, for panel (A) to (D).

**Figure 2:**
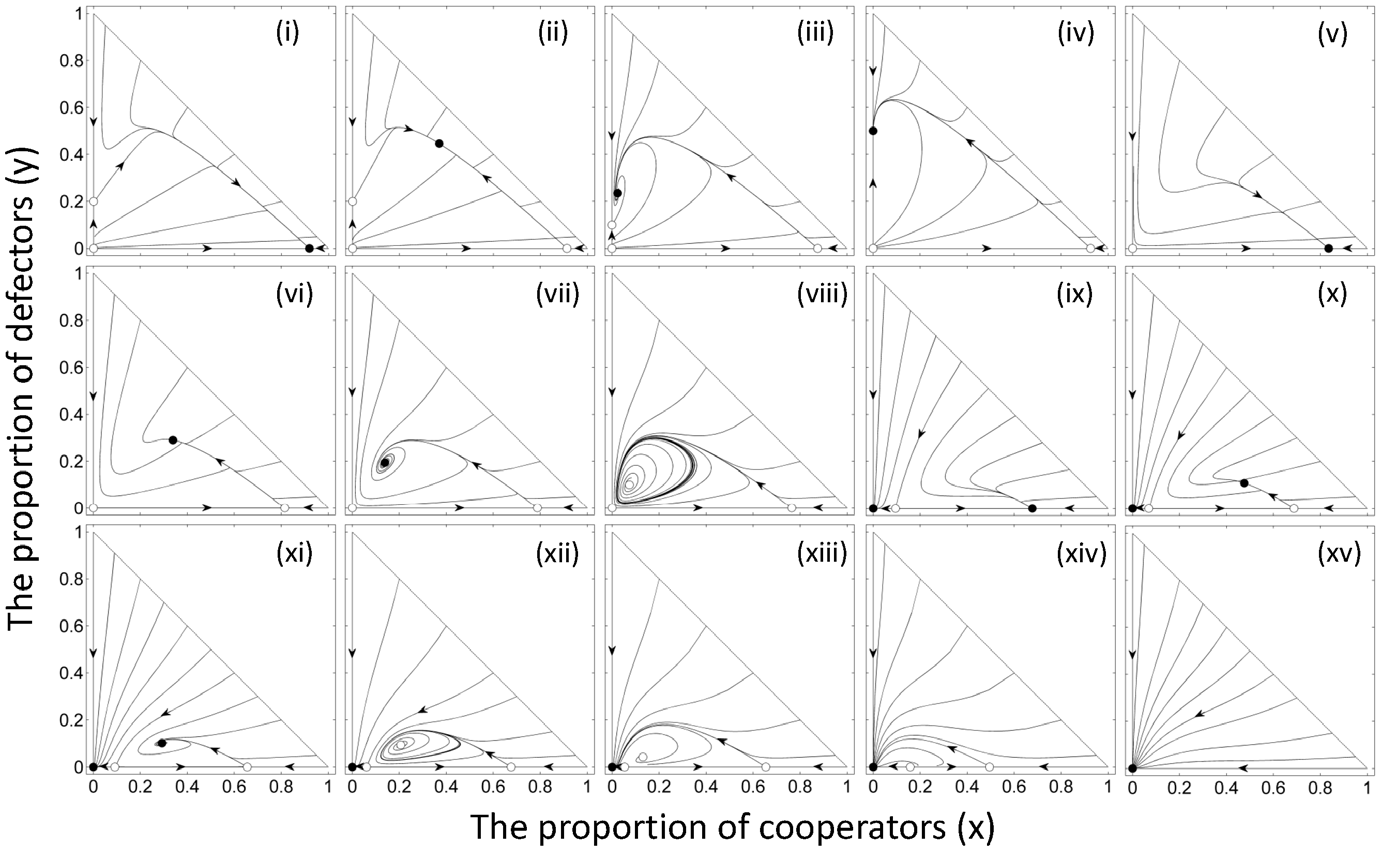
The behaviors of the dynamics of ecological prisoner's dilemma games on a phase plane. Plots (i)–(xv) correspond to part i–xv in Fig. 1, respectively. Solid circles represent stable equilibriums; open circles represent unstable equilibriums. Parameters are 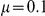, 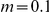, 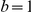 for all diagrams, except 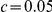 and 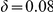 for (i), 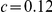 and 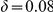 for (ii), 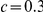 and 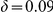 for (iii), 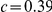 and 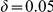 for (iv), 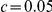 and 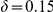 for (v), 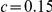 and 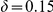 for (vi), 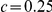 and 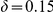 for (vii), 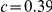 and 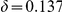 for (viii), 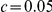 and 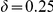 for (ix), 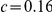 and 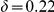 for (x), 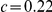 and 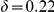 for (xi), 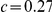 and 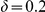 for (xii), 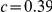 and 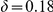 for (xiii), 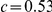 and 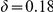 for (xiv), and 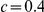 and 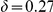 for (xv).

**Figure 3:**
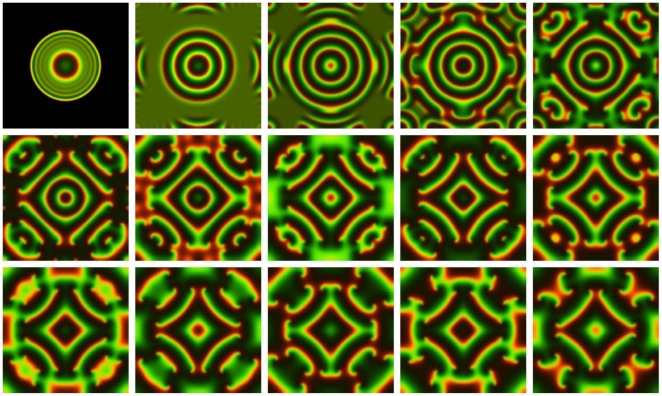
Spatial patterns of the ecological PDG on a 401×401 lattice. The color brightness indicates the probabilities that each site is occupied by a cooperator (red) or a defector (green), or remains empty (black) (as in Wakano et al. 2009). Initially, the central site is occupied by a cooperator with a probability of 0.1 or by a defector with the same probability, and the other sites are completely empty. Parameters are the same as Fig. 2 viii as for the dynamics of periodic oscillation. First snapshot is taken at time 1500; the others start from time 4500, with a temporal interval of 1500 time steps. See Animation S2 and Appendix S3 for details.

These boundary and interior equilibriums, as well as the bifurcation conditions, divide the parameter space into at most 15 different parts with the decrease of assortment, *m* (Figure 1); each part indicates a particular behavior of dynamics (Figure 2). Notably, the condition for cooperation invasion in frequency-dependent selection (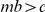) occupies only one part in the parameter space (left of the dashed lines in Figure 1). High death rate (mortality) and high cost to cooperate were shown to be important for generating damped oscillation (i.e. a focus; Figure 2 iii, vii, xi) and periodic oscillation (i.e. a limit circle; Figure 2 viii, xii) in population dynamics. A spatial simulation showed that the dynamics behavior of periodic oscillation can generate complicated spatial patterns (Figure 3; see Appendix S3 and Animation S2 for details). In the current density-dependent ecological PDG, parameters lie in the combined zone of the entire brown, yellow and cyan parts of Figure 1 can potentially lead to the evolution of cooperation. The condition of cooperation evolution, thus, has been largely expanded and relaxed in the ecological PDG; complex behaviors of dynamics can be expected.

## Results and Discussion

A social dilemma arises from the random interactions among individuals, and thus the key to unlocking this puzzle lies in creating nonrandom assortative interactions [16], [17]. Because the relatedness between players can be depicted by the difference between the probability for a cooperator to have a game with another cooperator and the probability for a defector to have a game with a cooperator (Appendix S1), the existence of assortative interactions becomes essential for the relatedness being positive. Our analysis thus confirmed the importance of assortment in facilitating the invasion and persistence of cooperation in ecological evolutionary games.

Moreover, low population density reduces the random encounter probability and exploitation from defectors; it further increases the relatedness being greater than the assortment (*m*) and thus favors the evolution of cooperation in the prisoner's dilemma game. This is consistent with Hauret et al.'s [42] results that cooperation can be promoted when there is a decrease of population density in the ecological public goods game. Evidence from Australian mountain possums indeed shows a significant negative relationship between the relatedness and the availability of local tree dens [59]. A low population density can reduce the exploitation by defectors and thus mitigate the tragedy of commons [60]. Therefore, factors that can reduce population density, such as empty sites [35], habitat saturation [53], [61], enhanced predation risk [62], habitat deterioration [37], [38] and fragmentation [39], provide potential solutions to the social dilemma.

This model is distinct from those models for viscous populations (an alternative way to consider ecological dynamics) [51], [52], [53] in two aspects: (i) in our model natal dispersal distance of offspring when colonizing empty sites is much longer than the interacting range between gaming individuals as confirmed in many animals (e.g. [45], [46], [47], [48], [49], [50]), whereas viscous population models consider a similar distance of natal dispersal to the interacting (or gaming) range; (ii) there is cost to an individual in the viscous populations even when surrounded only by empty sites, in contrast to no cost in our model. Evidently, the viscous populations could be certain bacterial strains that interact through the diffusion and absorption of biochemical products generated through metabolism, whereas our model is more suitable for depicting social animals (e.g. mate competition and coalition between male lions [63]).

The Hamilton rule provides a fundamental condition for the evolution of cooperation: the cooperative behavior can be favored if the benefit to cooperate discounted by the relatedness between the actor and recipient is still higher than the cost to cooperate [12], [18]. Variants of this rule have been illustrated under various evolutionary mechanisms [17], [19], [51], [53]. Assortative interactions between individuals play an important role in leading to the relatedness between players and are thus essential for the Hamilton rule [16], [17].

Population density can also affect the Hamilton rule by mediating the relatedness between players and the benefit and cost to cooperate, as in the ecological games of viscous populations [51], [52], [53]. However, in these models for viscous populations, the empty site is an implicit player: cooperators surrounded by empty sites only pay tribute yet without return and thus lose against empty sites. In our model, games only happen between individuals; there will be neither benefit nor cost for individuals surrounded only by empty sites. A comparison of these two kinds of models (see Appendix S4) suggests cooperation evolves easier in games only between individuals (our model) than games where cooperators compulsorily pay cooperative cost to the surrounding regardless of whether the surrounding is empty or not (as in the model for viscous populations [51], [53]).

When the baseline birth rate is less than the death rate, a defective population is incapable of surviving in the absence of cooperators. In contrast, a cooperative population can be sustained in such harsh environments by compensating the deficit in the population growth rate with the benefit gained from mutual cooperation. Parallel evidence is rich in ecology, showing that positive interactions between species (e.g. facilitation) prevail in stress environments, such as in desert and inter-tidal zones [64], [65], [66]. This result further suggests an alternative evolutionary sequence, in contrast to the one in classic evolutionary games. Individuals are often thought to be selfish initially in classic evolutionary games, while mutualism evolves afterwards. Here, we suggest an alternative evolutionary sequence in harsh environment: cooperation (symbiosis or mutualism) first appears in harsh environment, followed by the invasion of defection, which then inevitably leads to a common tragedy, or the social dilemma [67], [68]. The pioneer species that colonizes a barren habitat, as in the studies on community succession, are often symbiotic or social, followed by exploiters (competitive species) [69], [70].

In conclusion, for the evolution of cooperation, assortative interaction is crucial [16], [71], whereas harsh environments that causes a high death rate and low population density can also serve as an inducement for the cooperation evolution. Individuals in harsh environment are prone to be cooperative in order to combat the high death rate, with the cooperation benefits. Adding ecological dynamics into evolutionary games opens a much wider window for the evolution of cooperation, and thus exhibits a variety of dynamical behaviors.

## Supporting Information

Appendix S1 The derivation of the relatedness.
(DOC)

Appendix S2 The estimation of the critical states for bifurcations (including *MATLAB* code).
(DOC)

Appendix S3 The probability transition model for spatial dynamics of the ecological Prisoner's dilemma game (including *MATLAB* code).
(DOC)

Appendix S4 A comparison with the Hamilton rule for the viscous populations.
(DOC)

Animation S1 The behaviors of the dynamics of ecological prisoner's dilemma games on a phase plane.
(MP4)

Animation S2 Spatial patterns of the ecological prisoner's dilemma games.
(MP4)
