# Supplementary material for: Eco-Evolutionary Feedback and the Invasion of Cooperation in Prisoner's Dilemma Games
Source: PLoS One. 2011 Nov 18;6(11):e27523. doi: 10.1371/journal.pone.0027523 (PMC3220694; doi:10.1371/journal.pone.0027523)
Supplement: Appendix S1 — The derivation of the relatedness. (DOC) [file pone.0027523.s001.doc]

**Appendix S1: The derivation of the relatedness**

In the main text, $f_{C|C}$ is the probability that a given cooperative individual interacts with another cooperative individual; $f_{C|D}$ is the probability that a given defective individual interacts with a cooperative individual. We have the formula for the relatedness:

$r=f_{C|C}-f_{C|D}=\frac{f_{C}f_{C|C}}{f_{C}}-\frac{f_{D}f_{C|D}}{f_{D}}=\frac{f_{CC}}{f_{C}}-\frac{f_{DC}}{f_{D}}=\frac{f_{D}f_{CC}-f_{C}f_{DC}}{f_{C}f_{D}}$ (S1)

where $f_{\Omega}$ is the proportion of Ω-type (*C* or *D*) individuals in the population and $f_{\Omega\Omega'}=f_{\Omega}f_{\Omega'|\Omega}$ is the proportion of interactions between Ω- and Ω’-type individuals. Because there are only two strategies (*C* and *D*) in the population, we have $f_{C}+f_{D}=1$ and $f_{C}=f_{CC}+f_{DC}$, from which we can further obtain the following:

$r=\frac{f_{D}f_{CC}-f_{C}f_{DC}}{f_{C}f_{D}}=\frac{(1-f_{C})f_{CC}-f_{C}(f_{C}-f_{CC})}{f_{C}(1-f_{C})}=\frac{f_{CC}-f_{C}^{2}}{f_{C}(1-f_{C})}$. (S2)

Note that $f_{CC}-f_{C}^{2}$ is the covariance between the states of the actor and recipient, and $f_{C}(1-f_{C})$ is the variance of actor states. Moreover, considering the states of the actor and recipient as random variable *X* and *Y* (if the actor is a cooperator, *X*=1, otherwise *X*=0; so does the state of the recipient, *Y*=1 or 0). Clearly, the variance of *X* is $Var(X)=f_{C}(1-f_{C})$, and the covariance between *X* and *Y* is $Cov(X,Y)=E(XY)-E(X)E(Y)=f_{CC}-f_{C}^{2}$, where *E* denotes statistical expectation. Therefore, the relatedness in our model is actually the covariance between the states of two gaming individuals divided by the variance of the actor states, $r=Cov(X,Y)/Var(X)$.
