# Supplementary material for: Eco-Evolutionary Feedback and the Invasion of Cooperation in Prisoner's Dilemma Games
Source: PLoS One. 2011 Nov 18;6(11):e27523. doi: 10.1371/journal.pone.0027523 (PMC3220694; doi:10.1371/journal.pone.0027523)
Supplement: Appendix S2 — The estimation of the critical states for bifurcations (including MATLAB code). (DOC) [file pone.0027523.s002.doc]

**Appendix S2: The estimation of the critical states for bifurcations.**

The ecological prisoner’s dilemma game (equation (2)) only has one equilibrium for the coexistence of cooperators and defectors when the inequality (6) in the text holds. This interior equilibrium can be derived by letting the right-hand side of equation (2) equal zero:

. (S1)

The Jacobian matrix of ($\hat{x},\hat{y}$) is given by

(S2)

where and . Thus, we can get the characteristic equation as follows,

$\lambda^{2}-Tr\lambda+D=0$ (S3)

where is the trace of the Jacobian matrix, and $D=bxy(1-x-y)(1-m)[c(1-m)(1-x-y)+c_{x}-c_{y}]$ its determinant. Three kinds of bifurcations can be identified for equation (2) (Nayfeh & Balachandran 1995). First, the equilibrium (S1) changes from a stable node to a focus when the eigenvalue of the Jacobian matrix (the roots of characteristic equation (S3)) change from real number to imaginary number. That said, the critical point of the bifurcation is determined by $Tr^{2}-4D=0$. Specifically, this equation can be rewritten as follows:

$\begin{matrix} c^{2}(1-m)^{2}(4c^{2}b^{2}m^{2}-4cb^{3}m^{2}+4b^{4}m^{2}-4cb^{3}m-4c^{3}bm+c^{4}+2c^{3}b+c^{2}b^{2})\delta^{2} \\ +2c(1-m)(c-mb)^{2}(2b^{4}m^{2}-2cb^{3}m^{2}+2b^{3}m\mu-2cb^{3}m+2b^{2}c^{2}m-b^{2}c\mu\\ +2bc^{2}m\mu-2b\mu c^{2}-c^{3}\mu)\delta+\mu(c-mb)^{4}[4mb^{2}(b-c)+\mu(b+c)^{2}]=0 \end{matrix}$ (S4)

Second, a Hopf bifurcation that incurs a limit circle occurs when the trace of the Jacobian matrix disappears, $Tr=0$, and therefore we have

$c(1-m)(c-2mb)\delta-\mu(c-mb)^{2}=0$ (S5)

Third, when there were two boundary equilibriums for cooperators, a heteroclinic bifurcation occured once the limit circle touched the boundary equilibriums. It is difficult to deduce the critical point analytically, but we present here a numerical method using MATLAB (MathWorks, Inc.).

**References**

Natfeh, A.H., Balachandran, B., 1995. Applied nonlinear dynamics: analytical, computational, and experimental methods. John Wiley & Sons, New York.

***MATLAB code for Figure 1***

syms c delta x

Md=0.35;b=1;mu=0.1;m=0.1;

[C,D]=solve(delta-(c-m*b)*(m*b^2-c*b*m+c*mu)/((c^2)*(1-m)),delta-...

((b-c+mu)^2)/(4*(1-m)*(b-c)),delta,c);

C=double(C);D=double(D);

c1=linspace(0,C(2),100);

d1=((b-c1+mu).^2)./(4*(1-m)*(b-c1));

c2=linspace(m,1,100);

d2=mu*(c2-b*m)./(c2*(1-m));

c3=linspace(0,C(2),100);

d3=(mu+m*(b-c3));

cc=[c1,c3(end:-1:1)];

dd=[d1,d3(end:-1:1)];

fill(cc,dd,'cyan')

cc=[c3,1,0];

dd=[d3,mu,mu];

hold on

fill(cc,dd,'yellow')

cc=[c2,0,0];

dd=[d2,mu,0];

fill(cc,dd,'red')

ezplot(delta-mu*(c-b*m)/(c*(1-m)),[0,1,0,Md]);

ezplot(delta-(c-m*b)*(m*b^2-c*b*m+c*mu)/((c^2)*(1-m)),[0,1,0,Md]);

ezplot((b^2)*(m^2)*mu+2*b*c*m*delta-...

2*b*c*(m^2)*delta-2*b*m*mu*c+(c^2)*mu-...

(c^2)*delta+(c^2)*delta*m,[C(1),C(2),0,Md]);

f=(c^2)*((1-m)^2)*((2*c*b*m)^2-4*c*(b^3)*(m^2)+...

4*(b^4)*(m^2)-4*c*(b^3)*m-4*(c^3)*b*m+c^4+...

2*(c^3)*b+(c^2)*(b^2))*delta^2+...

2*c*(1-m)*((c-m*b)^2)*(2*(b^4)*(m^2)-...

2*c*(b^3)*(m^2)+2*(b^3)*m*mu-2*c*(b^3)*m+...

2*(b^2)*(c^2)*m-(b^2)*c*mu+2*b*(c^2)*m*mu-...

2*b*(c^2)*mu-(c^3)*mu)*delta+...

mu*((c-m*b)^4)*(4*m*(b^2)*(b-c)+mu*(b+c)^2);

ezplot(f,[0,C(1),0,Md]);

ezplot(f,[C(1),1,0,mu]);

plot([0,1],[0.1,0.1])

plot([m,m],[0,Md],':')

plot([0,0],[0,Md])

plot([0,1],[Md,Md])

% The folowing is the code to estimate heteroclinic bifurcation

cc=linspace(min(C),max(C),50);

dd=zeros(size(cc));

y=0;

for l=1:length(cc)

c=cc(l);

delta1=(mu+m*(b-c));

delta2=((b-c+mu)^2)/(4*(1-m)*(b-c));

while abs(delta2-delta1)>0.0001

delta=(delta1+delta2)/2;

ff=(mu+(m+(1-m)*x)*(b-c)-(1-m)*y*c)*(1-x-y)-delta;

X=solve(ff,x);

X=double(X);

T=20000;

xx=zeros(1,T);yy=zeros(1,T);

xx(1)=max(X);

yy(1)=0.005;

for t=1:T-1

xx(t+1)=xx(t)+xx(t)*((mu+(m+(1-m)*xx(t))*(b-c)...

-(1-m)*yy(t)*c)*(1-xx(t)-yy(t))-delta);

yy(t+1)=yy(t)+yy(t)*((mu+(1-m)*xx(t)*b)*(1-xx(t)-yy(t))-delta);

end

if sqrt(xx(end)^2+yy(end)^2)<0.0001

delta2=delta;

elseif sqrt((min(C)-mean(xx(T-18000:T)))^2 ...

+(mean(yy(T-18000:T)))^2)>0.0001

delta1=delta;

else

dd(l)=delta;

break

end

end

dd(l)=(delta1+delta2)/2;

end

plot(cc,dd)

hold off

axis([0,1,0,Md])

axis square

xlabel('The cost-to-benetif ratio (c/b)')

ylabel('The mortality-to-benetif ratio (c/b)')

title('')
