# Supplementary material for: Eco-Evolutionary Feedback and the Invasion of Cooperation in Prisoner's Dilemma Games
Source: PLoS One. 2011 Nov 18;6(11):e27523. doi: 10.1371/journal.pone.0027523 (PMC3220694; doi:10.1371/journal.pone.0027523)
Supplement: Appendix S3 — The probability transition model for spatial dynamics of the ecological Prisoner's dilemma game (including MATLAB code). (DOC) [file pone.0027523.s003.doc]

**Appendix S3: The probability transition model for spatial dynamics of the ecological Prisoner’s dilemma game.**

The proportion of cooperators ($x$) and defectors ($y$) in the ecological Prisoner’s dilemma game can be interpreted as the probabilities that a site is occupied by a cooperator or by a defector (Wakano et al. 2009). We limit the game and the colonization through reproduction within the player’s neighboring sites. The dynamics of the occupancy probability for a specific site can be defined by the following ordinary differential equation (Zhang et al. 2006),

$\begin{matrix} \frac{dx_{i}}{dt}=\frac{1}{n}\sum(c_{xj}x_{j})(1-x_{i}-y_{i})-\delta x_{i} \\ \frac{dy_{i}}{dt}=\frac{1}{n}\sum(c_{yj}y_{j})(1-x_{i}-y_{i})-\delta y_{i} \end{matrix}$ (S1)

where $x_{i}$ and $y_{i}$ represents the probability that site $i$ is occupied by cooperators and defectors, $n$ the number of sites in the neighborhood of site $i$, $c_{xj}$ and $c_{yj}$ is colonization rate of cooperators and defectors in the neighboring site $j$. The colonization rates are given as follows,

$\begin{matrix} c_{xj}=\mu+\left(m+(1-m)\frac{1}{n}\sum x_{k}\right)(b-c)-(1-m)\frac{1}{n}\sum y_{k}c \\ c_{yj}=\mu+(1-m)\frac{1}{n}\sum x_{k}b \end{matrix}$ (S2)

Here, notation represents the sum of relevant quantities in the neighborhood. This model belongs to the extended cellular automata and has widely been applied in the pattern formation of spatial ecology (e.g. Li et al. 2005). The MATLAB code (MathWorks, Inc.) is provided below.

**References**

Li, Z.Z., Gao, M., Hui, C., Han, X.Z., Shi, H., 2005. Impact of predator pursuit and prey evasion on synchrony and spatial patterns in metapopulation. Ecol. Model. 185: 245-254.

Wakano, J.Y., Nowak, M.A., Hauert, C., 2009. Spatial dynamics of ecological public goods. Proc. Natl. Acad. Sci. USA 106: 7910-7914.

Zhang, F., Li, Z.Z., Hui, C., 2006. Spatiotemporal dynamics and distribution patterns of cyclic competition in metapopulation. Ecol. Model. 193: 721-735.

***MATLAB code for the spatial simulation***

% **************** Parameter values and variables ****************

T=2000; % Total time step

n=401; % Lattice size

b=1;c=0.39; % benefit and cost

mu=0.1;delta=0.137; % baseline birth rate and mortality

m=0.1; % Assortment

X=zeros(n+2,n+2); % Occupancy probability matrix

Y=zeros(n+2,n+2);

XX=zeros(n+2,n+2); % Tempera matrix

YY=zeros(n+2,n+2);

X((n+3)/2,(n+3)/2)=0.1; % Initial proportion of cooperators

Y((n+3)/2,(n+3)/2)=0.1; % Initial proportion of defectors

Cx=zeros(n+2);Cy=zeros(n+2); % Payoff matrix

XY=zeros(n,n,3); % Image matrix

% ****************************************************************

F=figure('position',[100 100 200 200]);

M=moviein(T);

for t=1:T

for k=1:2

X(1,:)=X(n+1,:);X(n+2,:)=X(2,:);

X(:,1)=X(:,n+1);X(:,n+2)=X(:,2);

Y(1,:)=Y(n+1,:);Y(n+2,:)=Y(2,:);

Y(:,1)=Y(:,n+1);Y(:,n+2)=Y(:,2);

SX=(X(2:n+1,1:n)+X(2:n+1,3:n+2)...

+X(1:n,2:n+1)+X(3:n+2,2:n+1))/4;

SY=(Y(2:n+1,1:n)+Y(2:n+1,3:n+2)...

+Y(1:n,2:n+1)+Y(3:n+2,2:n+1))/4;

Cx(2:n+1,2:n+1)=mu+(m+(1-m)*SX)*(b-c)...

-(1-m)*SY*c;

Cy(2:n+1,2:n+1)=mu+(1-m)*SX*b;

Cx(1,:)=Cx(n+1,:);Cx(n+2,:)=Cx(2,:);

Cx(:,1)=Cx(:,n+1);Cx(:,n+2)=Cx(:,2);

Cy(1,:)=Cy(n+1,:);Cy(n+2,:)=Cy(2,:);

Cy(:,1)=Cy(:,n+1);Cy(:,n+2)=Cy(:,2);

XC=Cx.*X;YC=Cy.*Y;

SCx=(XC(2:n+1,1:n)+XC(2:n+1,3:n+2)...

+XC(1:n,2:n+1)+XC(3:n+2,2:n+1))/4;

SCy=(YC(2:n+1,1:n)+YC(2:n+1,3:n+2)...

+YC(1:n,2:n+1)+YC(3:n+2,2:n+1))/4;

XX(2:n+1,2:n+1)=X(2:n+1,2:n+1)...

+SCx.*(1-X(2:n+1,2:n+1)-Y(2:n+1,2:n+1))...

-delta*X(2:n+1,2:n+1);

YY(2:n+1,2:n+1)=Y(2:n+1,2:n+1)...

+SCy.*(1-X(2:n+1,2:n+1)-Y(2:n+1,2:n+1))...

-delta*Y(2:n+1,2:n+1);

X=XX;Y=YY;

end

XY(:,:,1)=X(2:n+1,2:n+1);

XY(:,:,2)=Y(2:n+1,2:n+1);

MX=max(XY(:));

imagesc(XY/MX);

axis square

axis off

figure(F);

M(t)=getframe;

end

movie2avi(M,'movie')
