# Supplementary material for: Eco-Evolutionary Feedback and the Invasion of Cooperation in Prisoner's Dilemma Games
Source: PLoS One. 2011 Nov 18;6(11):e27523. doi: 10.1371/journal.pone.0027523 (PMC3220694; doi:10.1371/journal.pone.0027523)
Supplement: Appendix S4 — A comparison with the Hamilton rule for the viscous populations. (DOC) [file pone.0027523.s004.doc]

**Appendix S4:** **A comparison with the Hamilton rule for the viscous populations.**

Here, we present a model of ecological games where individuals not only interact with each other but also pay cost to empty sites (i.e. the empty site is an implicit player), as in the models for viscous populations (van Baalen & Rand, 1998; Le Galliard et al., 2003; Lion & Gandon, 2009). Following the definition of assortment in the text, an individual interacts with individuals of the same strategy at a probability of *m*, and randomly encounters other individuals and empty sites at a probability of 1-*m*. Thus, in the viscous population with a proportion of *x* cooperators and *y* defectors, the mean payoff for a cooperator is $(m+(1-m)x)b-c$ (different from the mean payoff in our model) and for a defector $(1-m)xb$. If the birth rates of cooperator and defector are given by

$\begin{matrix} c_{x}=\mu+(m+(1-m)x)b-c \\ c_{y}=\mu+(1-m)xb \end{matrix}$, (S1)

and we further assume the same death rates for cooperative and defective individuals. The condition for cooperation to increase in a population can be obtained by the inequality $c_{x}>c_{y}$:

$rB>C$. (S2)

Here, $B=b$ and $C=c/q_{+|+}$ where $q_{+|+}=m+(1-m)(x+y)$ is the probability that a given individual (C or D) encounters another individual (not an empty site), and $r=f_{C|C}-f_{C|D}$ is still the relatedness (see Appendix 1) where $f_{C|C}=[m+(1-m)x]/[m+(1-m)(x+y)]$ and $f_{C|D}=(1-m)x/[m+(1-m)(x+y)]$. This Hamilton rule S2 is distinct from the Hamilton rule eqn 4 in the main text by the exact form of its cost *C*. Clearly, the value of *C* (=*c*/*q*_+|+_, where *q*_+|+_<1) in eqn S2 is greater than the value of *C* (=*c*) in the text. This shows that cooperation can be evolved more easily in games between individuals (as the one in the text) than games with empty sites acting as an implicit player which incurs a high cost load.

**References**

Le Galliard L, Ferriere R, Dieckmann U (2003) The adaptive dynamics of altruism in spatially heterogeneous populations. Evolution 57: 1-17.

Lion S, Gandon S (2009) Habitat saturation and the spatial evolutionary ecology of altruism. J. Evol. Biol. 22: 1487-1502.

van Baalen M, Rand RA (1998) The unit of selection in viscous populations and the evolution of altruism. J. Theor. Biol. 193: 631-648.
